# New York Odontological Society

**Published:** 1894-01

**Authors:** 

**Affiliations:** New York Odontological Society


### Reports of Society Meetings.

### NEW YORK ODONTOLOGICAL SOCIETY.

A REGULAR meeting of the New York Odontological Society was held on Tuesday evening, October 17, 1893, at the New York Academy of Medicine, No. 17, West Forty-third Street, New York City, with the vice-president, Dr. Brockway, in the chair.

The minutes of the previous meeting were first read and approved.

### INCIDENTS OF OFFICE PRACTICE AND CASUAL COMMUNICATIONS.

Dr. Perry showed some new corundum wheels, which had been sent to him by Dr. Darby's assistant.

*Dr. Mitchell*, of London.—I have a new method of attaching a porcelain face to a pivot, where the face has been broken off, leaving the pivot *in situ* and in a solid condition, and where it would be inadvisable to remove it, with the collar or pin perfectly firm and merely the face broken off. The method of repairing it is to cut the pin off, and grind it down, and with a separating file or (as I prefer) a small circular saw in the engine, cut down to where the pin originally was, making a forked arrangement for the pin to set down in. Adjust the porcelain face in the desired position, and with a small amount of modelling composition take an impression of the back of the tooth with the pins running through the backing as far as possible, and let the impression material run over the point of the new face. Take it off, and pour carefully, so as to get a good impression, then remove, and you have the tooth set up in its new position. Then make use of an excavator or a sharp-pointed knife to cut away a portion of the model; and take a piece of very soft platinum and anneal it, cutting a couple of holes for the pins to go through, and making a groove near the base of the old backing so that the edge of the platinum may come down; leaving it in the condition so that when flush it can be finished up nicely. Stick together with stick-wax the backing and the tooth, remove carefully and invest in plaster, and sand, or marble-dust, filling the inside perfectly, and finish as usual. It is one of those little things in practice that help us considerably, for we all know the difficulty of attaching a porcelain face to a back and pivot in position. It is also a practical operation. I can show you one that was broken off twice through accident after being in place for a year. It was broken through occlusion on the stem of a pipe, while playing polo. The second time the accident not only broke off the tooth, but also wrenched off a portion of the pin. I forgot to mention that finally the crown is set with cement.

Dr. Van Woert then read the paper of the evening.

(For Dr. Van Woert's paper, see page 30.)

DISCUSSION.

*Dr. Perry.*—Will you define a little more definitely one point you mention? You spoke of deposits on the roots of teeth, and also of micro-organisms. You guard against the danger of infection, which implies the presence of pathogenic germs. Will you state more distinctly your theory?

*Dr. Van Woert.*—I am under strong conviction this disease is infectious, and is conveyed from one mouth to another through carelessness in sterilizing instruments, and the second application of the same piece of dam, as it is impossible to sterilize rubber dam without spoiling. It should never be used but once.

*Dr. Hill.*—What is your theory in regard to the loss of the bone?

*Dr. Van Woert.*—I believe the bone is absorbed in the same way that it is carried off after the extraction of a tooth.

*Dr. Perry.*—I like to see a man have a theory. I am given to having theories, and mine have been partly the same as Dr. Van Woert expressed this evening. I am not so sure about the infection, nor about the calcareous deposit being always primarily the cause. I think micro-organisms have much to do with it. I expressed my opinion before this Society ten years ago, in a paper on the treatment of the roots of teeth, when I said that teeth were lost by the encroachment of bacteria, caused primarily and principally by the accumulation of tartar, although tartar is not always the chief cause, for Dr. Tomes has shown, and many of us know, that sometimes teeth come out free from tartar. I have never ventured to undertake the Riggs operation of scraping the bone to any extent, for I have never seen the evidence that would convince me that his theory was true. The accumulations of tartar under the margin of the gum and the insidious action of pathogenic germs I think combine to do the harm. Many people who are affected with phthisis have this disease of the teeth. We know that the teeth of some people who are red-blooded and healthy do no not loosen though they have large accumulations of tartar, while in some people who are also seemingly healthy the teeth do loosen and finally drop out free from calcic deposits. How do you explain it? What connection there is between the loosening of the teeth and pulmonary troubles I do not know; but some day I believe we will find that bacteria are important factors in producing the condition. I think this Society is to be congratulated upon the starting of this plan of conservative treatment around the roots of teeth instead of the radical treatment that we have used heretofore. I believe that with all the remedies I have used I have accomplished more by the use of Buchan's carbolic soap and listerine than anything else I know of for lesions about the necks of the teeth. If we could induce our patients to daily clean out the pockets under the margin of the gum by the use of the syringe and prevent fermentation, it would help considerably; but there are other things to do in the world, and it is difficult to induce every one to use it. I think, as Dr. Van Woert said, the disease cannot be cured, although it can be alleviated. I do not believe in medicines as a rule, but I have seen as much good from the use of Buchan's soap as any remedy I have ever used.

*Dr. Littig.*—Dr. Perry says patients cannot carry around syringes, but they do have their fingers with them, and I have obtained better results by directing patients to rub their fingers on the gums, and using listerine, than with anything else. Many cases have been kept in good condition in that way. You do not always need the syringe to remove the food deposited in those pockets. The constant accumulation of food and of micro-organisms continues the disease, and the use of the fingers to rub the gums I find to be an excellent substitute for the syringe.

*Dr. Davenport.*—Will Dr. Van Woert kindly inform me whether I understood him correctly in advising the removal of only so much of the calculus with instruments as can be reached without laceration of either the gum or the peridental membrane? Secondly, I understood him to say that, instead of removing the calculus underneath the gum with scalers, even though the membrane has ceased to exist upon the root of the tooth where the pocket was, he applies an acid which so softens the deposit that it can be easily rinsed out. Considering the similarity between tartar and the inorganic portion of a tooth, is there not great danger of injury to the root by the use of an acid powerful enough to quickly (it must be quickly, as he says care should be taken to keep the saliva away) dissolve that substance? Will not the acid probably make sensitive afterwards the periphery of the live root?

*Dr. Van Woert.*—I think Dr. Davenport misunderstood me. I first remove all the deposits possible with the scalers. Upon careful examination it will be found that these deposits are never in direct contact with the membrane; they are thrown up a little above, and if care is taken in using the scalers they can be removed without injury to the membrane. The question of keeping the pockets clear of saliva is not a very grave one, for with napkins and saliva-pump it is an easy matter to keep them clear for an hour if necessary. It takes very little trichloracetic acid to soften these deposits, and the effect is more than pleasing. You need have no fear of a slough, and can rely on the gums assuming their normal appearance in a few days. I use it a great deal with very gratifying results.

*Dr. Davenport.*—What effect has it on the cementum?

*Dr. Van Woert.*—None that I can see, and Professor Peirce claims the same. I would say to Dr. Perry, I believe there are deposits in every case; while they are not always thoroughly calcified, they are there and they break down the union between the membrane, the tooth, and the alveolus, generally making more rapid inroads on the buccal than on the lingual surfaces.

*Dr. Watkins.*—What solution of the trichloracetic acid do you use?

*Dr. Van Woert.*—Almost saturated. I get an ounce bottle of the crystals and fill it with water. This makes a very satisfactory solution. Dr. Kirk, of Philadelphia, has used it, and he claims even more than I do for it. Drs. Hill and Brockway have also used it with satisfaction.

*Dr. Parker.*—I wish to make a little plainer than perhaps Dr. Van Woert did the question asked by Dr. Davenport. A year and a half ago the question he asked gave me trouble relative to the use of the trichloracetic acid. The first case I tried it on was in my own family. My wife had a tooth which I had been treating for some time, and I concluded to try the acid. That was about fourteen months ago. I used two or three applications, and the tooth is now entirely healthy. I have tried it in a number of cases, and upon teeth freshly extracted, to see if I could notice any effect, and I have never been able to see any harm. I am a great believer in trichloracetic acid.

*Dr. Mitchell.*—What is its action?

*Dr. Parker.*—There is no penetration. It acts on the surface.

*Dr. Mitchell.*—Is there any difference between the trichloracetic and chromic acids?

*Dr. Parker.*—I have never used chromic acid, and cannot say.

*Dr. Howe.*—I would like to record the statement that I have seen many cases of so-called pyorrhœa alveolaris not accompanied by calcareous deposits. I have two cases in hand now, in which considerable absorption of the alveolar borders has taken place, and from the beginning I have been unable to find any calcareous deposits under the gum margin. I have for many years made no attempt whatever to scrape the margins of the alveolar border, for I have not believed that necrosis existed in the cases that I have had to treat. Necrosis of bone is so different in its manifestations from what has appeared in my cases that I have been unable to understand some of the statements and methods of others. I have never seen even the slightest symptoms or developments indicating caries or necrosis of the alveolar processes. No bony sequestra or even granular particles of bone have ever appeared, nor have I been able to discover the bare surface of the alveolar borders with suitable probes, as I would expect to do if either of these diseased conditions were in progress. I congratulate Dr. Van Woert on the ability with which he has presented what I must regard as nearly correct views; and also for the moral courage with which he has taken this position.

Dr. Van Woert, I believe, expressed the opinion that the disease was caused by the calcareous deposit, or that the deposits are a factor in producing it. From my observation of cases, I have regarded a calcareous deposit as merely an accompaniment of some cases, increasing the trouble but not causing it. In others the deposit may approach more nearly to being a factor in its origin. Dr. Black has used the term "phagedenic pericementitis." That expresses to me the idea of a pericemental inflammation which results in destruction, and Dr. Van Woert's suggestion of the method of the alveolar walls disappearing appears very plausible. It seems to me, however, that the appearance or non-appearance of calcareous deposits on the roots of teeth is mostly an incident,—a much larger factor in keeping up the diseased process than in originating it. And it seems, also, that micro-organisms are over-rated in importance as factors in producing the lesions we are considering. Even if it should be shown that it were possible to carry the disease from one mouth to another by infection, it would not, to my mind, prove that the disease was caused primarily and solely by the micro-organisms, without a predisposition already existing. I think the time will come when the stress laid upon micro-organisms as causative factors will not be made so emphatic as it is now.

About two years ago Dr. Littig spoke to me of finger massage of the gums, accompanied by the use of stimulating lotions, such as listerine, being useful in the treatment of this trouble, and I have advised many people to pursue that practice, with decided benefit. I think it is an aid towards restoring an approach to normal circulation in the tissue.

*Dr. Perry.*—I cannot agree with Dr. Howe that the time for pathogenic germs has passed by.

*Dr. Howe.*—I did not say that.

*Dr. Perry.*—Perhaps I ought to be more explicit. I spoke to a gentleman who had spent three or four years in Europe studying medicine, and he said the scientific men there were becoming more and more emphatic in their opinion that many diseases are caused by micro-organisms.

*Dr. Howe.*—Was he talking of pyorrhœa alveolaris?

*Dr. Perry.*—He was talking of diseases, and if that is not one I do not know what it is. Many years ago I did a great deal of work with the microscope. I made, for quite a number of years, many careful examinations of all material I could find under the margin of the gum, and the number of micro-organisms present there was remarkable and produced the conviction that they were important factors. Of course, I do not mean that pyorrhœa can be cured when it has its origin through heredity. The hair cannot be prevented from turning gray, nor can the wrinkles on the face be smoothed when they appear. Neither can this disease in all cases be cured. In many persons it is incident to middle life and old age. It never will be cured while human nature is what it is. Several years ago, Dr. F. Y. Clark, on the floor before this Society, had a great deal to say about bacteria as important factors in producing decay of the teeth. He was very much laughed at. Is there any doubt to-day about their influence in caries of teeth? Not that they originate decay, for that is not claimed, but when the enamel is dissolved by acid action, and they can gain access to the dentine, they then become active factors in destroying the structure. And so I believe it is with the loosening of the teeth. When the gum becomes detached, whether by tartar or by a loss of tone or vitality of the gum or of the general system, so that a pocket is formed, micro-organisms find shelter and by their insidious presence become active factors in hastening the disease. How else can one account for the loosening and final loss of a tooth here and there, while the adjoining teeth in the same mouth stand firm and untouched.

*Dr. Howe.*—That is the opinion that I hold. If micro-organisms are the cause of this disease, then the conclusion would be that it is purely local. If you can sterilize the margins of the gums and the pockets, you will stop the disease and cure it right then and there. If it is a local disease only, patients that are regularly in our hands have a right to hold us responsible if the disease ever makes any progress. But if micro-organisms are rather an incident or development of the disease, which is primarily due to systemic conditions, then destruction of the micro-organisms will not necessarily cure the disease, although I grant it may be an essential prerequisite to recovery.

*Dr. Jarvie.*—This is one of the most interesting and important subjects we could possibly discuss. I believe that in the practice of every gentleman present teeth in the mouths of patients over twenty-five years of age are lost more frequently from this disease than from all others put together. I certainly have, in the last five years, given it more attention than any other class of cases that come under my care, and while I think I know more about it to-day than I did, I do not know as much in regard to the cause as I would like, or as to the best means of treatment. I do think I have made progress. The principal feature, as I understand it, in the paper presented to-night is the statement in opposition to the generally-accepted one that no necrosis, as a rule, is present in pyorrhœa alveolaris. It is generally accepted from the teachings received in past years that necrosis is almost always present. For several years I have been an examiner in the Board of Censors in this State on this disease and its treatment, and I believe that nine out of ten of those who come before me tell me that one of the conditions of this disease is necrosis of the alveolus. I have never seen it, and for years I have been very firmly convinced that it is not a condition of pyorrhœa alveolaris. If this be true, what are we to think of the statement of Dr. Riggs and many other intelligent men, that in treating this disease they trim away and remove the *necrosed* borders of the alveolus? It has been stated here that there are almost as many cases of pyorrhœa alveolaris where deposits of lime were not present as there were cases where it was present. In all my practice I have seen but one case of pyorrhœa alveolaris where there was no deposit of tartar on the root. Judging from Dr. Howe's description, I should infer that he had classed as cases of pyorrhœa alveolaris those I do not regard as such,—recession of the gums and of the process, but no inflammation and no pus.

*Dr. Howe.*—Have you tested those cases with peroxide of hydrogen to see whether there was any reaction?

*Dr. Jarvie.*—No; but in a case upon which I have been working for the last two weeks the gums are just as hard as can be and quite free from inflammation or congestion, and yet on the superior cuspids the gums have receded one-half the length of the root, and there is not a particle of pus. It is simply a wasting away of the substance, and not only of gum tissue, but there is much erosion upon twelve of the upper and ten of the lower teeth,—that is, twenty-two teeth out of the thirty-one that were in the mouth. There is so much erosion that the pulps are almost exposed. I do not think, as a rule, that we find erosion and pyorrhœa affecting the same teeth.

*Dr. Perry.*—Would you call the case you have described one of pyorrhœa?

*Dr. Jarvie.*—No; but I thought that possibly Dr. Howe would class that as pyorrhœa. I do not find any serumic calculus in those cases, so that possibly we misunderstand each other by the terms we use. My mode of treatment has been almost identical with that described by Dr. Van Woert,—the removal of the deposit as much as possible, using pyrozone to wash out the pockets, following that with the use of trichloracetic acid. While I have not experimented with trichloracetic acid out of the mouth, I have in the mouth. In one case where my scalers seemed to make but little impression on the deposit, I bathed the tooth with the acid for one, two, or three minutes, and, while this alone did not take it off, it softened it so I could remove it much more easily. Unfortunately this deposit will not always locate where it is readily reached, and sometimes it will be in spots where it is almost impossible for any human being to remove it. I have seen one or two cases of pyorrhœa alveolaris that I considered absolute cures, but those were the only ones.

*Dr. Perry.*—Was the treatment the same?

*Dr. Jarvie.*—No; the cases occurred before I knew anything about pyrozone or trichloracetic acid. One was a case that I commenced about twelve years ago, and the other ten years. The treatment was simply the thorough removal of the deposits and the use of aromatic sulphuric acid. Undoubtedly there have been constitutional changes in the patient. I believe that the disease itself is largely due to constitutional tendencies. In nearly all the most obstinate cases we find that the patients have hard, dense teeth, as though there were an excess of lime about the mouth, and the most valuable teeth are the ones that are lost by this disease. I cannot believe that the micro-organisms are the *cause* of this disease. I find they are an important factor in the spread of it. Where pockets are formed and places of lodgement for food are made you will have micro-organisms, and the disease will increase much more rapidly under that condition; therefore the necessity is still more urgent for antiseptic treatment. The pyrozone and the trichloracetic acid will bring about a thorough antiseptic condition. You never will bring a case to anything like a satisfactory condition unless you have the full assistance of the patient, and, unfortunately, in many cases you do not get it. Often, after doing the best we are capable of, we see the patient after one or two weeks, only to be discouraged by the continued negligence and indifference manifested. The patients may say they have cleaned their teeth three or four times a day, while the fact remains that they have not *cleaned* them at all, although they may have brushed them. There is a vast difference, sometimes, between brushing and cleansing the teeth.

*Dr. Howe.*—Will Dr. Jarvie repeat what he said about erosion? I did not understand what erosion had to do with the condition of the absorbed border.

*Dr. Jarvie.*—I do not think it has any relation. I simply spoke of it. There was no deposit of lime in the case I mentioned, and no exudation of pus; and I spoke of the teeth being all eroded.

*Dr. Perry.*—I repeat that I do not mean that micro-organisms are the only cause of this trouble. I stated that I believed they are a very important factor. There must be a letting down of the vital forces. I believe that when they get an opportunity they are most powerful factors. Do not understand me as saying that germs begin the trouble, for I do not hold to that view. It is easy to be misunderstood, and this only shows how careful one ought to be in statement.

*Dr. Jarvie.*—Almost every speaker to-night, except myself, has said that he had never seen a case of pyorrhœa cured. That necessarily implies that none of the speakers has ever effected a cure. I wish we had some of the gentlemen here who have accomplished this, that they might tell us of their mode of treatment, for there are dentists who tell their patients that they can cure most of the cases, and some dentists in New York assert that in this age of skill and intelligence it is not necessary that any teeth should be lost through the ravages of this dread disease. I had hoped that we might have heard from such, and gained such wisdom and knowledge from them that I at least might have so profited that I should no more see tooth after tooth, sound and free from decay, in the mouths of some patients, loosening slowly but surely, and patient and myself powerless to do aught but retard and not arrest the disease.

*Dr. Mitchell.*—I want to endorse what was said about dead bone. There is something so characteristic about dead bone that there is no mistake about it if it be present. I wished to speak of another matter, and that is that in those cases where we find quite an amount of absorption of the bone, without any appreciable absorption of the gum, we find pockets that are moderately deep, extending below where the union would be between the tooth and the peridental membrane almost to the extremity of the roots. We find those pockets filled with a fluid of some kind that undoubtedly must have the power of breaking down the bone, melting it and consequently modifying the support of the tooth. That will give an acid reaction. The fact of its persistent presence there gradually but surely breaks down the bony support of the tooth. It need not be very much acidified to do so. It is a fluid that has a peculiar organization, and the capacity for producing a retrograde condition of the parts is most marked. I have had a number of those cases, and they were rather difficult to clear up. I find that something of a stimulating nature is the best thing to use. I have used carbolic acid and caustic potash, and also nitrate of silver, with very marked success. In regard to cases where we have a great deal of pus, I regard hydrogen peroxide as unreliable for the purpose of showing us true pus or giving us a true pus reaction. The reaction of peroxide of hydrogen is almost identical in fresh blood as with pus. I have tried it in a number of cases, and it is so similar that it is very difficult to observe any difference. Therefore if any statements were made in regard to its being a good indicator of pus they should be taken with some grains of allowance.

*Dr. Howe.*—Perhaps I ought to say that in using the term "pyorrhœa alveolaris," I do not apply it to that form of absorption of gum and alveolar borders which is evidently due to deposits of salivary calculus, but restrict it to those cases in which the alveolar borders are wasted so that pockets are formed of more or less depth, which tend constantly to increase and extend. There is not always a perceptible amount of pus present, but generally more or less makes its appearance if the gum is squeezed under the finger. If scales or granules of calculus are present on the root, the gum ordinarily has a congested appearance; but in those cases which have no hard deposits on the root the gum is generally anæmic in appearance and looks shrunken where the alveolus is wasted beneath it. I think these distinctive appearances are generally, although not always, present in what I suppose is usually known as pyorrhœa alveolaris.

*Dr. Perry.*—Do you consider pyorrhœa alveolaris an inherited disease?

*Dr. Howe.*—Decidedly,—a disease that may be inherited and often is.

*The Chairman.*—Before we adjourn, I wish on behalf of the Society to thank Dr. Van Woert and those gentlemen who participated in the discussion this evening.

Adjourned.

John I. Hart, D.D.S.,
*Editor New York Odontological Society.*

